# Lecanemab Treatment for Alzheimer's Disease in Real‐World Settings in Eastern China

**DOI:** 10.1002/alz70857_102024

**Published:** 2025-12-25

**Authors:** Yanxing Chen, Siyan Zhong, Xinyi Zhang

**Affiliations:** ^1^ Department of Neurology, the Second Affiliated Hospital, School of Medicine, Zhejiang University, Hangzhou, Zhejiang, China

## Abstract

**Background:**

Lecanemab, an anti‐amyloid antibody, has been introduced for the treatment of Alzheimer's disease (AD) since June 28, 2024 in China. However, experiences with lecanemab in Chinese population is lacking. This report presents the real‐world findings from early AD patients receiving lecanemab in eastern China.

**Method:**

We prospectively collected data from early AD patients who were treated with lecanemab. The Appropriate Use Recommendations for lecanemab were employed to guide patient selection, as well as the detection and management of amyloid‐related imaging abnormalities (ARIA).

**Result:**

Among the 60 patients treated, infusion reactions were observed in 13 individuals, manifesting as fever (10 cases), rash (2 cases), headache (2 cases), insomnia (1 case). ARIA was detected in 4 out of 42 patients who received at least five infusions. Notably, none of these ARIA cases were symptomatic. Specifically, one case with both moderate ARIA‐E and H was observed prior to the 5^th^ infusion. Another 3 cases of ARIA (1 moderate ARIA‐H, 1 moderate ARIA‐E, 1 mild ARIA‐H) were identified before the 7^th^ infusion among 35 patients. No further ARIA occurrences were observed in the 11 patients tested before the 14^th^ infusion.

**Conclusion:**

These data support that ARIA occurrs early and is usually asymptomatic, higlighting the importance of early monitoring in clinical practice.

